# Effects of salinity on the treatment of synthetic petroleum-industry wastewater in pilot vertical flow constructed wetlands under simulated hot arid climatic conditions

**DOI:** 10.1007/s11356-020-10584-8

**Published:** 2020-09-01

**Authors:** Thomas V. Wagner, Fatma Al-Manji, Jie Xue, Koen Wetser, Vinnie de Wilde, John R. Parsons, Huub H. M. Rijnaarts, Alette A. M. Langenhoff

**Affiliations:** 1 Department of Environmental Technology, Wageningen University & Research, P. O. Box 17, 6700 EV Wageningen, The Netherlands; 2 Institute for Biodiversity and Ecosystem Dynamics (IBED), University of Amsterdam, P. O. Box 94248, 1092 GE Amsterdam, The Netherlands

**Keywords:** Benzotriazole, Benzoic acid, Zinc, *Phragmites australis*, *Typha latifolia*, Vertical flow constructed wetlands

## Abstract

Petroleum-industry wastewater (PI-WW) is a potential source of water that can be reused in areas suffering from water stress. This water contains various fractions that need to be removed before reuse, such as light hydrocarbons, heavy metals and conditioning chemicals. Constructed wetlands (CWs) can remove these fractions, but the range of PI-WW salinities that can be treated in CWs and the influence of an increasing salinity on the CW removal efficiency for abovementioned fractions is unknown. Therefore, the impact of an increasing salinity on the removal of conditioning chemicals benzotriazole, aromatic hydrocarbon benzoic acid, and heavy metal zinc in lab-scale unplanted and *Phragmites australis* and *Typha latifolia* planted vertical-flow CWs was tested in the present study. *P. australis* was less sensitive than *T. latifolia* to increasing salinities and survived with a NaCl concentration of 12 g/L. The decay of *T. latifolia* was accompanied by a decrease in the removal efficiency for benzotriazole and benzoic acid, indicating that living vegetation enhanced the removal of these chemicals. Increased salinities resulted in the leaching of zinc from the planted CWs, probably as a result of active plant defence mechanisms against salt shocks that solubilized zinc. Plant growth also resulted in substantial evapotranspiration, leading to an increased salinity of the CW treated effluent. A too high salinity limits the reuse of the CW treated water. Therefore, CW treatment should be followed by desalination technologies to obtain salinities suitable for reuse. In this technology train, CWs enhance the efficiency of physicochemical desalination technologies by removing organics that induce membrane fouling. Hence, *P. australis* planted CWs are a suitable option for the treatment of water with a salinity below 12 g/L before further treatment or direct reuse in water scarce areas worldwide, where CWs may also boost the local biodiversity.

**Electronic supplementary material:**

The online version of this article (10.1007/s11356-020-10584-8) contains supplementary material, which is available to authorized users.

**Figure:**
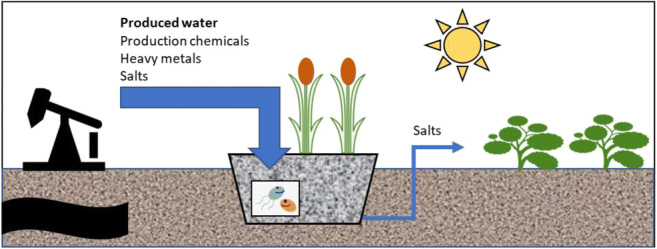
Graphical abstract

## Introduction

Oil extraction generally results in the production of extensive volumes of petroleum industry wastewater (PI-WW) that contains heavy oil fractions and light hydrocarbons (Fakhru’l-Razi et al. 2009). Heavy oil fractions are commonly separated from PI-WW aboveground in oil/water separators (Murray-Gulde et al. 2003). The remaining water contains light hydrocarbons, heavy metals, salts, and conditioning chemicals, such as corrosion inhibitors and antiscalants (Fakhru’l-Razi et al. 2009; Rehman et al. 2018; Afzal et al. 2019), and its specific composition depends on the characteristics of the oil reservoir and the conditioning chemicals that were used in the oil extraction process (Ozgun et al. 2013). After the oil/water separation, there are different options for further management of PI-WW: injection back into the subsurface formation where the oil was extracted from, discharge aboveground, or reuse in the oil extraction process (Fakhru’l-Razi et al. 2009). However, since oil extraction often occurs in water-scarce locations, such as the Arabian peninsula, it is beneficial to treat and reuse the PI-WW instead of disposing of it to enhance the local water supply. In areas with sufficient water supply, the treatment and reuse, instead of disposal of PI-WW, protects the environmental quality of surface water and prevents the contamination of groundwater that is potentially used as a drinking water source. Various physical, chemical, and biological treatment technologies for PI-WW that could allow its reuse have been studied in recent years (Fakhru’l-Razi et al. 2009; Sosa-Fernandez et al. 2018, 2019; Sudmalis et al. 2018). Amongst these, nature-based treatment in the form of a constructed wetland (CW) is an attractive treatment option, because it has low energy and maintenance requirements, it is regarded as aesthetically enriching landscapes, and it can function as an oasis in desert landscapes where it can significantly improve the local biodiversity (Stefanakis 2018, 2019, 2020a).

CWs are man-made wetland systems in which various natural processes remove contaminants from a water stream, such as adsorption, biodegradation, photodegradation, and plant uptake (Garcia et al. 2010; Wagner et al. 2018; Hashmat et al. 2018). These simultaneously working removal mechanisms allow the treatment of water streams with a wide variety of contaminants. In fact, one of the largest CWs in the world was built in the desert of Oman for the treatment of PI-WW (Stefanakis et al. 2018). The treatment of PI-WW in CWs results in the removal of heavy oil fractions and grease compounds (Abed et al. 2014; Alley et al. 2013; Horner et al. 2012; Ji et al. 2002, 2007; Stefanakis 2018a, b), light hydrocarbons (Stefanakis 2020b), heavy metals (Alley et al. 2013; Horner et al. 2012; Ji et al. 2002, 2007; Kanagy et al. 2008), organics (Ji et al. 2002, 2007; Stefanakis 2018, 2019; Rehman et al. 2019) and total nitrogen (Ji et al. 2002, 2007, Stefanakis 2018, 2019) from the PI-WW with a low salinity. The removal of conditioning chemicals from PI-WW in CWs has not been assessed yet. In addition, it is not known what the effect of increasing salinities of PI-WW on the treatment efficiency of the CW is. The salinity of PI-WWs ranges worldwide from 1 to 300 g/L and is the result of both subsurface geochemical conditions and the injection of oil recovery fluids (Rosenblum et al. 2017; Al-Ghouti et al. 2019). Although the PI-WW does show temporal changes in composition and salinity (Rosenblum et al. 2017), the dominant ions in PI-WW are generally sodium and chloride (Fakhru’l-Razi et al. 2009). The salinity determines the viability of plants in the CW (Klomjek and Nitisoravut 2005) and influences removal mechanisms, such as biodegradation and adsorption (Wagner et al. 2018). Furthermore, the salinity of the CW effluent will determine the options for reuse of the treated PI-WW.

In the present study, synthetic PI-WW with light hydrocarbons and increasing salinities was treated in lab-scale vertical-flow CWs planted with *Phragmites australis* or *Typha latifolia* under hot arid conditions to assess the effect of an increasing salinity on the viability of *P. australis* and *T. latifolia*. In addition, we determined the impact of an increasing salinity on the removal of a heavy metal (zinc), a conditioning chemical (benzotriazole) and an aromatic hydrocarbon (benzoic acid) from synthetic PI-WW, and the contribution of the plant species to the removal of these pollutants.

## Materials and methods

### Chemicals

Calcium chloride dihydrate (CaCl_2_∙2H_2_O), di-potassium hydrogen phosphate trihydrate (K_2_HPO_4_∙3H_2_O), magnesium sulphate heptahydrate (MgSO_4_∙7H_2_O), urea, zinc chloride (ZnCl_2_) and yttrium standard solution Y(NO_3_)_3_ in HNO_3_, 1000 mg/L Y) were obtained from Merck KGaA (Darmstadt, Germany). Sodium chloride (NaCl), methanol (LC-grade) and acetic acid (99%) were obtained from VWR Chemicals (Leuven, Belgium). Benzoic acid and benzotriazole were obtained from Carl Roth GmbH (Karlsruhe, Germany).

Benzotriazole, benzoic acid, urea, CaCl_2_∙2H_2_O, K_2_HPO_4_∙3H_2_O, MgSO_4_∙7H_2_O, ZnCl_2_ and NaCl were used for the production of synthetic PI-WW. Acetic acid, methanol and Y(NO_3_)_3_ in HNO_3_ were used for sample preparation and analysis.

### Lab-scale constructed wetlands

Six vertical-flow CWs based on the CWs described by Saha et al. (2020) were built in glass containers of 25 × 25 × 25 cm with dark sides to prevent algae growth. The CW substrate consisted of 3 layers. The bottom layer contained 4 cm of 8–16 mm gravel (GAMMA, Wageningen, The Netherlands) for optimal water drainage. This was covered with a 16-cm layer of a mixture of 75% sand with a diameter of > 2 mm (GAMMA, Wageningen, The Netherlands) mixed with 25% sediment from a fully operational *P. australis* planted surface-flow CW to inoculate the substrate. This layer was covered with 2 cm of the 8–16 mm gravel for optimal water distribution. Two CWs were planted with *P. australis*, two CWs were planted with *T. latifolia* and two CWs remained unplanted to determine the effect of vegetation on the CW treatment efficiency. The CWs were placed in a Heraeus Vötsch MC 785-KLIMA climate chamber (Weiss Technik, Stuttgart, Germany) that simulated the climatic circumstances at the Arabic peninsula with a temperature of 30 °C, relative humidity of 57.2% and a light exposure time of 9.5 h/day. The CWs were fed 4 times per day, 45 min and 0.85 L per feeding cycle, by a Watson Marlow 205U pump (Rotterdam, The Netherlands), which resulted in a theoretical hydraulic retention time of approximately 1 day. The effluent was collected in 500-mL Schott bottles with a continuous overflow system to the sewer.

### Influent composition

The CWs were fed with a mix of synthetic PI-WW with light hydrocarbons and synthetic domestic wastewater (Table 1). A mixture of synthetic PI-WW with synthetic domestic wastewater was used to simulate mixing of these two water streams at oil extraction facilities, which could supply the plants in the CW with necessary nutrients. The composition of the synthetic municipal wastewater was based on the guidelines for synthetic wastewater preparation of the OECD test 303A (OECD 2001). In the synthetic PI-WW, benzoic acid was used as a representative aromatic hydrocarbon. Benzotriazole was added as representative conditioning chemical because of its use as corrosion inhibitor in the oil extraction process. Zinc is one of the more common heavy metals in PI-WW and originates partly from the galvanized steel structures used in oil extraction (Lee and Neff 2011). Sodium chloride was used to adjust the salinity of the synthetic wastewater mixture, since these ions are the most common ions in PI-WW (Fakhru’l-Razi et al. 2009; Jimenez et al. 2018; Al-Ghouti et al. 2019).

**Table 1:** Composition of synthetic mix of petroleum-industry wastewater and domestic waste water

Synthetic petroleum-industry wastewater components	Concentration	Synthetic domestic waste water components	Concentration
Benzoic acid	40 mg/L	Urea	30 mg/L
Benzotriazole	1 mg/L	K_2_HPO_4_∙3H_2_O	28 mg/L
ZnCl_2_	10 mg/L	CaCl_2_∙2H_2_O	4 mg/L
NaCl	2, 4, 8, 12 mg/L	MgSO_4_∙7H_2_O	2 mg/L

### Experimental timeline

During the first 10 days, the CWs were fed with only synthetic domestic wastewater to start up the microbial activity in the CWs. After 10 days, the systems were fed with the mixture of synthetic PI-WW and synthetic municipal wastewater with a salinity of 2 g/L. Sampling started 24 days later. The salinity of the synthetic mix was increased from 2 to 4, 8 and 12 g/L every 21 days.

### Sampling and sample preparation

Liquid samples were taken twice per week from the influent and effluent of the CWs. Samples for benzotriazole analysis were filtered over a 0.2-μm polyethersulfone (PES) filter, diluted 100× with ultrapure water and stored in 1.5-mL glass LC vials at − 20 °C until analysis. Samples for benzoic acid analysis were filtered over a 0.2-μm PES filter, acidified with 1% (v/v) acetic acid (99%) and stored in 1.5-mL glass LC vials at − 20 °C until analysis. Samples for zinc analysis were spiked with 1% (v/v) of the yttrium internal standard solution (“Chemicals” section).

### Analyses

The temperature, pH and electrical conductivity (EC) were measured with a Hach Lange HQ440D multimeter (Tiel, The Netherlands). Benzotriazole was analysed by LC-MS/MS as described by Wagner et al. (2020a). Benzoic acid was determined by HPLC-DAD as described by Wagner et al. (2020b). Zinc was measured based on a certified ICP method of the Belgian federal agency of food chain security (2017/I-MET-209/LAB/FLVVG) using a PerkinElmer Avio 500 ICP-OES spectrometer (Groningen, The Netherlands) in axial and radial plasma view mode. The high-energy (f/6.7) echelle-based ICP-OES utilized two SCD detectors covering the spectral range from 163 to 782 nm. The injector tube was made of alumina and had an internal diameter of 2 mm. Argon (99.998%, Linde Gas, The Netherlands) was used as the plasma (10 L/min), auxiliary (0.2 L/min) and nebuliser gas (0.6 L/min). Nitrogen (99.996%, Linde Gas, The Netherlands) was used as purging gas (2 L/min) in the optical system of the spectrometer. The ICP-OES was operated with a plasma power of 1300 W, a signal integration time of 5 s, and peak processing in peak area mode, using three points/peak for integration. The analytical wavelength of Zn(II) was 206.200 nm. The emission line of Y(II)-371.027 was used as an internal standard. Data acquisition and analysis were performed with Syngistix™ Atomic Spectroscopy Software.

## Results and discussion

### The influence of salinity on plant growth

*Typha latifolia* and *Phragmites australis* both grew well at a synthetic PI-WW salinity of 2 g/L and a temperature of 30 °C (Fig. S1), demonstrating that these plant species are suitable choices for CWs treating mildly saline water in hot, arid climates. A picture of the planted and unplanted CWs during the complete experimental period is provided in the supplementary info (SI) (Fig. S1). *P. australis* survived better with increasing salinities then *T. latifolia*, as shown by visual observations of the plant colour, plant height and plant leaf density. At a salinity of 4 g/L, the *T. latifolia* started to discolour, while no visual effect of the increased salinity on the *P. australis* was observed (Fig. S1). At a salinity of 8 g/L, the *T. latifolia* completely discoloured and died, while the growth of the *P. australis* seemed to be impeded and its leaves became a lighter tone of green (Fig. S1). At 12 g/L, *P. australis* leaves discoloured and the plant height decreased (Fig. S1).

The impact of the salinity on *P. australis* has already been studied extensively, and *P. australis* is known to tolerate salinity levels below 15 g/L, with a higher tolerance for rhizome-grown plants, as used in the present study, than for seed-grown plants (Lissner and Schierup 1997). *P. australis* is relatively tolerant to NaCl stress because it restricts the transport of Na^+^ and Cl^−^ ions to the leaves, it intrinsically enhances the water use efficiency and it produces proline to adjust the intracellular osmotic pressure (Pagter et al. 2009). A too high salt concentration reduces the biomass of *P. australis* as a result of osmotic and ion-specific effects (Pagter et al. 2009), as was observed at a NaCl concentration of 12 g/L in the present study. The impact of the salinity on plant growth is less extensively studied for *T. latifolia* compared to *P. australis*. *T. latifolia* is reported to grow well in CWs treating shrimp farming wastewater with a salinity of 3–8 g/L, where the *T. latifolia* overgrew 9 other planted species in the second year of growth (Tilley et al. 2002). In addition, *T. latifolia* grew well in CWs treating fish farming wastewater with a salinity of 24 g/L (Jesus et al. 2014), which contradicts with the complete withering of the *T. latifolia* at a NaCl concentration of 8 g/L in the present study. The difference in salinity tolerance of *T. latifolia* in the present study and the study of Jesus et al. (2014) can be the result of a difference in origin of the plants, a difference in the climatic circumstances in which the plants were grown, or a different effect of the type of wastewater. Hootsman and Wiegman (1998) observed that *T. latifolia* died as result of treatment with 18 g/L sea salt that mainly contains NaCl, whereas *P. australis* was able to survive. For hot climatic conditions, when living vegetation is required in the CW, *P. australis* proves to be a better choice as vegetation in CWs treating saline water.

### The influence of constructed wetland treatment on the electrical conductivity

**Fig. 1:**
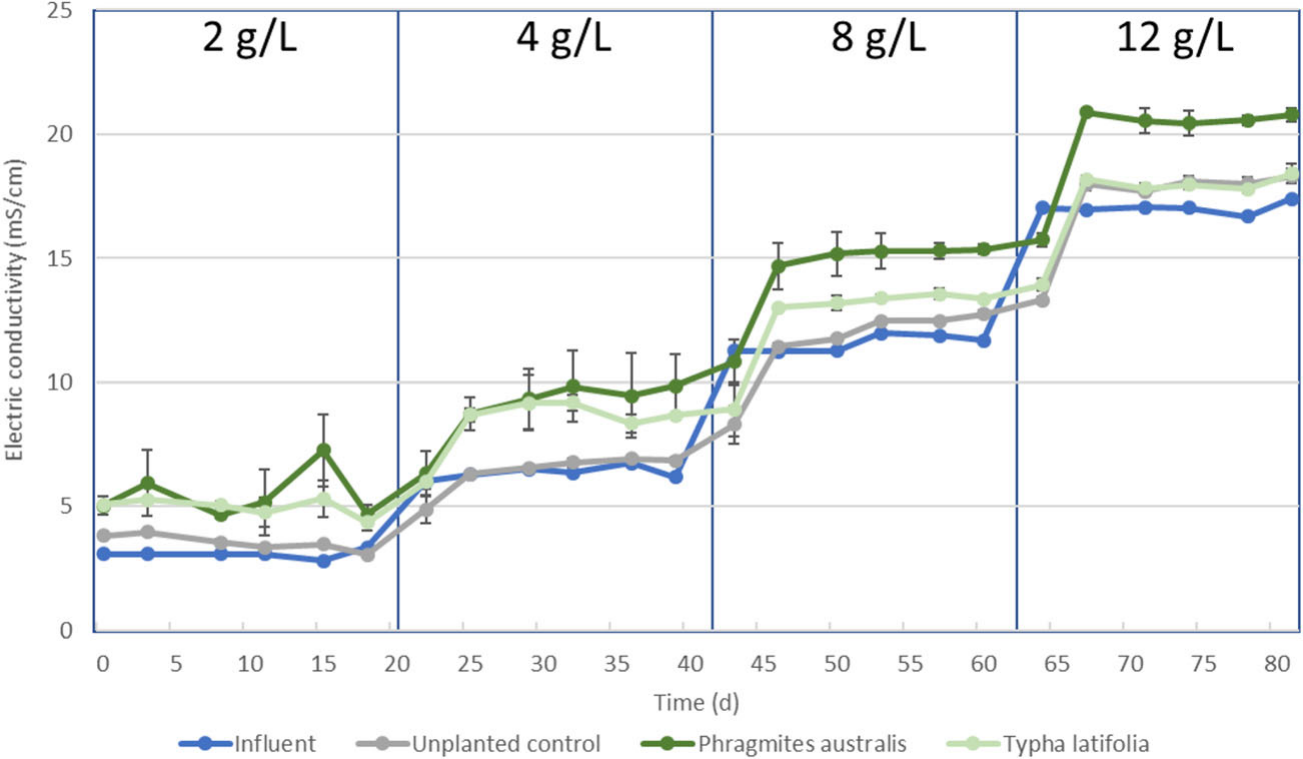
Influent and effluent electrical conductivity over time with increasing NaCl concentrations

The presence of *P. australis* and *T. latifolia* substantially impacted the electrical conductivity (EC) of the effluent of the CWs, as long as the plants were alive (Fig. 1).

A NaCl influent concentration of 2 g/L resulted in an EC of ~ 3 mS/cm, while a NaCl influent concentration of 12 g/L resulted in an EC of ~ 17 mS/cm (Fig. 1), suggesting that NaCl is the main contributing factor to the EC. The EC of the effluent of the unplanted CWs was slightly higher than the EC of the influent (Fig. 1), most likely due to evaporation of the water as a result of the high temperature. The presence of *P. australis* and *T. latifolia* substantially increased the EC of the treated effluent as a result of plant transpiration (Fig. 1). Combined evaporation and plant transpiration, evapotranspiration, resulted in an effluent EC that was substantially higher than the influent EC during low salt concentrations of 2 g/L and 4 g/L for both plant species (Fig. 1). The EC of the effluent increased because plants protect their plant tissue against toxic concentrations of mainly Na^+^ and Cl^−^ ions by excluding these when they take up water with their root system (Munns and Tester 2008), leaving these ions in the water that exits the CW as effluent.

The visual observations of the plant vitality (“The influence of salinity on plant growth” section) are corroborated by the trends of the EC of the effluent of the planted CWs. With a NaCl concentration of 2 g/L, the EC of the effluent of the CWs planted with *P. australis* and *T. latifolia* is similar to each other (Fig. 1), revealing a similar evapotranspiration rate. During the addition of 4 g/L NaCl, the EC of the *P. australis* planted CW effluent was higher than the EC of the *T. latifolia* planted CW effluent. The difference in EC between the two vegetation types further increased when the salt concentrations were increased to 8 g/L and 12 g/L NaCl, demonstrating a higher evapotranspiration in the *P. australis* planted CWs. During the increase of the NaCl concentration from 4 to 12 g/L, the EC of the effluent of the *T. latifolia* planted CWs approached the EC of the effluent of the unplanted CWs, illustrating that plant transpiration stopped. This is in agreement with visual observations of the *T. latifolia* death (“The influence of salinity on plant growth” section). The higher EC of the *P. australis* effluent compared to the unplanted CWs at 12 g/L NaCl implies that the plant still actively transpires water and is thus functional, despite the (partial) leaf decolouration (“The influence of salinity on plant growth” section).

### The impact of an increasing salinity on the removal of benzoic acid

**Fig. 2:**
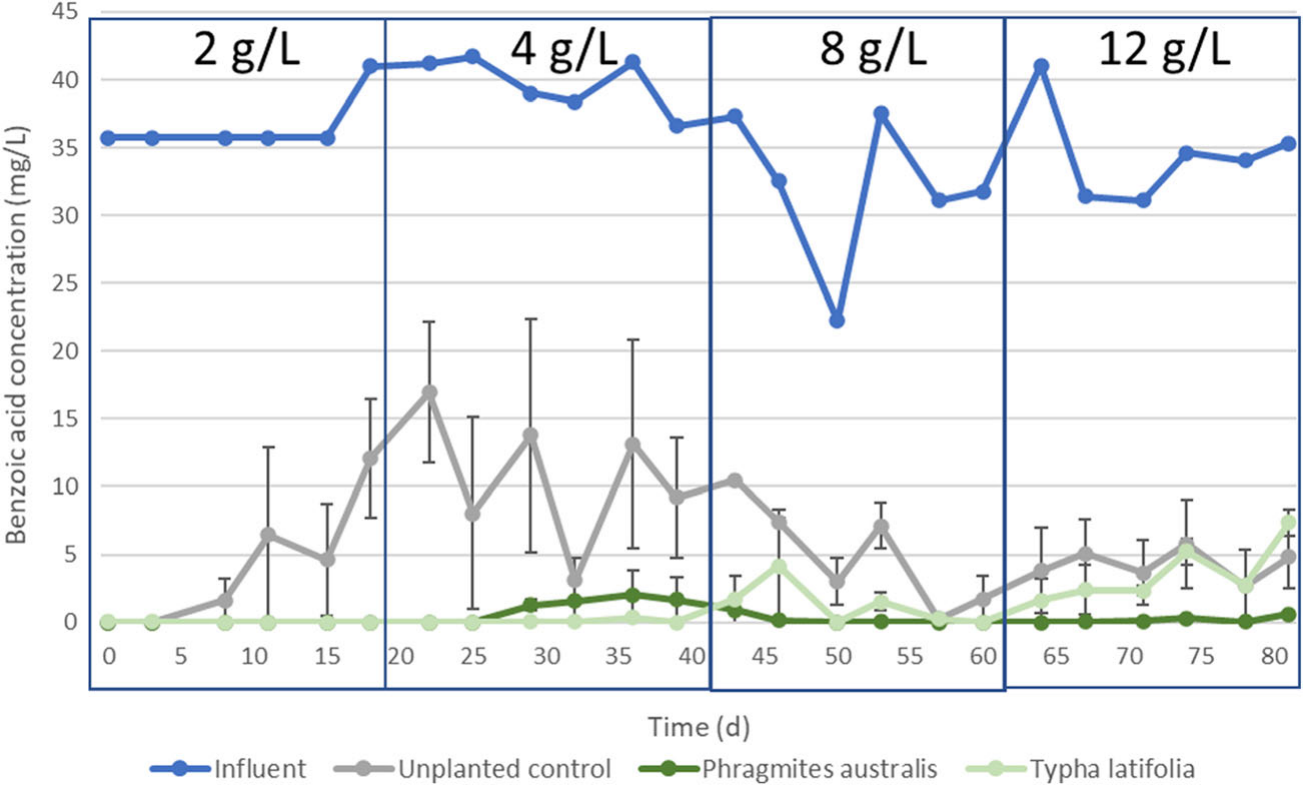
Influent (blue line) and effluent benzoic acid concentrations over time with increasing NaCl concentrations in *P. australis* planted CWs (dark green line), *Typha latifolia* planted CWs (light green line) and unplanted CWs (grey line). The effluent of both the *Phragmites australis* planted CWs and *Typha latifolia* planted CWs were 0 during the first 25 days and thus overlap

Aromatic hydrocarbon benzoic acid was completely removed in planted CWs at a NaCl concentration of 2 g/L, as demonstrated by the low residual concentrations in the effluent of the CWs compared to the influent concentration (Fig. 2). Complete benzoic acid removal in planted CWs was observed previously (Zachritz et al. 1996; Wagner et al. 2020c) and was the result of biodegradation (Wagner et al. 2020b). Previous studies also showed that biodegradation was the main removal mechanism in subsurface-flow CWs for other aromatic hydrocarbons, such as phenol (Stefanakis et al. 2016). Benzoic acid biodegradation occurs both in aerobic and anaerobic conditions, and many microorganisms, such as *Pseudomonas*, can degrade benzoic acid (Ziegler et al. 1987; Parsons et al. 1988; Hall et al. 1999). *Pseudomonas* was also identified by Hashmat et al. (2018) in their surface-flow CWs treating water contaminated with aromatic hydrocarbons. Benzoic acid did not adsorb to CW substrate (Wagner et al. 2020b). Plant uptake of benzoic acid cannot be ruled out in the present study, but the benzoic acid removal efficiency of the unplanted CWs varied between 70 and 100% at a NaCl concentration of 2 g/L (Fig. 2), which was solely the result of biodegradation. Hence, biodegradation is the main removal mechanism for benzoic acid in the present study and complete benzoic acid removal in the planted CWs is probably because the presence of vegetation results in a higher microbial density and microbial activity (Gagnon et al. 2007; Zhang et al. 2010; Nguyen et al. 2019), since the plant rhizosphere provides nutrients and favourable redox conditions (Gagnon et al. 2012). In the unplanted CWs, the absence of the vegetation probably results in less favourable conditions for the microorganisms, resulting in a decreased benzoic acid removal efficiency after the first 5 days.

With an increasing NaCl concentration increasing to 8 g/L, benzoic acid remains almost completely removed in the planted CWs (Fig. 2). Surprisingly, the removal efficiency in the unplanted CW increased over time (Fig. 2). This could be an indication that the microbial biomass in the newly started CWs increased despite the increasing NaCl concentrations, resulting in a higher benzoic acid removal efficiency. An increasing biodegradation efficiency with increased salinities is in contrast with Lin et al. (2008) and Villasenor Camacho et al. (2017), who observed decreasing biodegradation efficiencies in CWs fed with influent with increasing salinities. At a NaCl concentration of 12 g/L, the residual benzoic acid concentration in the effluent of the unplanted CWs stabilized at around 5 mg/L. The benzoic acid is still completely removed in the *P. australis* planted CWs (Fig. 2). In contrast, at this NaCl concentration, the benzoic acid concentration in the effluent of the *T. latifolia* planted CWs became similar to the effluent concentration of the unplanted CWs (Fig. 2). We hypothesize that the microbial community in this CW was less well able to cope with the increased NaCl concentration, as a result of the loss of its favourable environment due to the death of the *T. latifolia* (“The influence of salinity on plant growth” section). In the present study, a cutoff point for benzoic acid biodegradation was not observed for NaCl concentrations up to 12 g/L. Castillo-Carvajal et al. (2014) showed that aromatic hydrocarbons can be biodegraded by halophilic bacteria up to a NaCL concentration of 200 g/L.

### The impact of an increasing salinity on the removal of benzotriazole

**Fig. 3:**
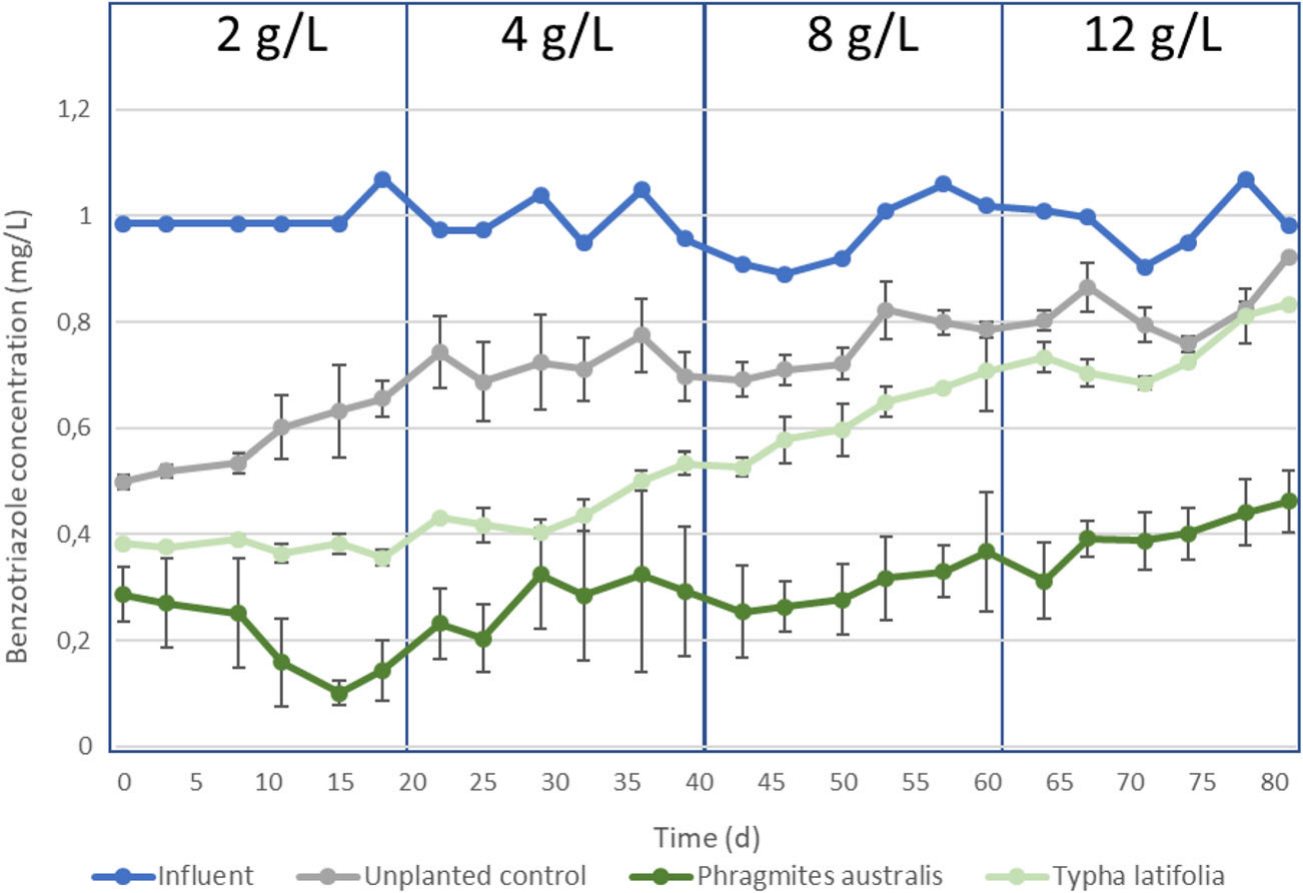
Influent (blue line) and effluent benzotriazole concentrations over time with increasing NaCl concentrations in *P. australis* planted CWs (dark green line), *Typha latifolia* planted CWs (light green line) and unplanted CWs (grey line)

The removal efficiency of the unplanted, *P. australis* planted and *T. latifolia* planted CWs for conditioning chemical benzotriazole differed substantially at a NaCl concentration of 2 g/L (Fig. 3).

The lowest benzotriazole removal efficiency at a NaCl concentration of 2 g/L was observed in the unplanted CWs (Fig. 3). The removal of benzotriazole in CWs is the result of simultaneous adsorption to the substrate and aerobic biodegradation, as demonstrated before (Wagner et al. 2020a). The presence of plants in the present study enhanced the benzotriazole removal efficiency, and the CWs planted with *P. australis* had a higher removal efficiency than the *T. latifolia* planted CWs (Fig. 3). The uptake and transformation of benzotriazole in the plants was not studied; hence, it is not possible to distinguish between direct enhancement as a result of plant uptake and degradation in the plant tissue and an indirect enhancement as a result of a more favourable environment by microorganisms in the plant rhizosphere. However, the uptake and degradation of benzotriazole by plants in CWs has been described for other plant species than those used in the present study, such as *Arabidopsis thaliana* and *Carex praegracilis* (LeFevre et al. 2015; Pritchard et al. 2018). The benzotriazole removal efficiency at a mild salinity of 2 g/L under hot arid circumstances in the *P. australis* planted vertical-flow CWs (Fig. 3) was comparable with the benzotriazole removal efficiency ranging from 63 to 90% in vertical-flow CWs treating domestic wastewater or cooling tower water in less hot climates as observed by others (Matamoros et al. 2010; Kahl et al. 2017; Brunsch et al. 2018; Nivala et al. 2019; Brunsch et al. 2020; Wagner et al. 2020a).

Increasing NaCl concentrations from 2 to 12 g/L lowered the benzotriazole removal efficiency in the planted and unplanted CWs (Fig. 3). However, despite the high evapotranspiration and corresponding concentration increase of both NaCl and fractions that are not taken up by the plants, there is still a net removal of benzotriazole over the whole experimental period for the planted and unplanted CWs (Fig. 3). The increasing difference between the *P. australis* planted CWs and *T. latifolia* planted CWs indicates that the death of the *T. latifolia* (“The influence of salinity on plant growth” section) is responsible for the large decrease in benzotriazole removal efficiency in the *T. latifolia* planted CWs. At a salinity of 12 g/L, the removal efficiency of the unplanted and *T. latifolia* planted CW was similar, indicating the absence of a positive effect of the vegetation on the benzotriazole removal in these CWs. In the *P. australis* planted CWs, the benzotriazole removal efficiency at a NaCl concentration of 12 g/L was slightly lower than at 2 g/L, but still showed more than 50% removal (Fig. 3). Given the benzotriazole removal efficiency difference between the *P. australis* and *T. latifolia* planted CWs, this is the result of biological removal with *P. australis,* and *P. australis* is still capable of stimulating the biological benzotriazole removal at a NaCl concentration of 12 g/L, despite the first signs of plant discolouration (“The influence of salinity on plant growth” section).

### The impact of an increasing salinity on the removal of zinc

**Fig. 4:**
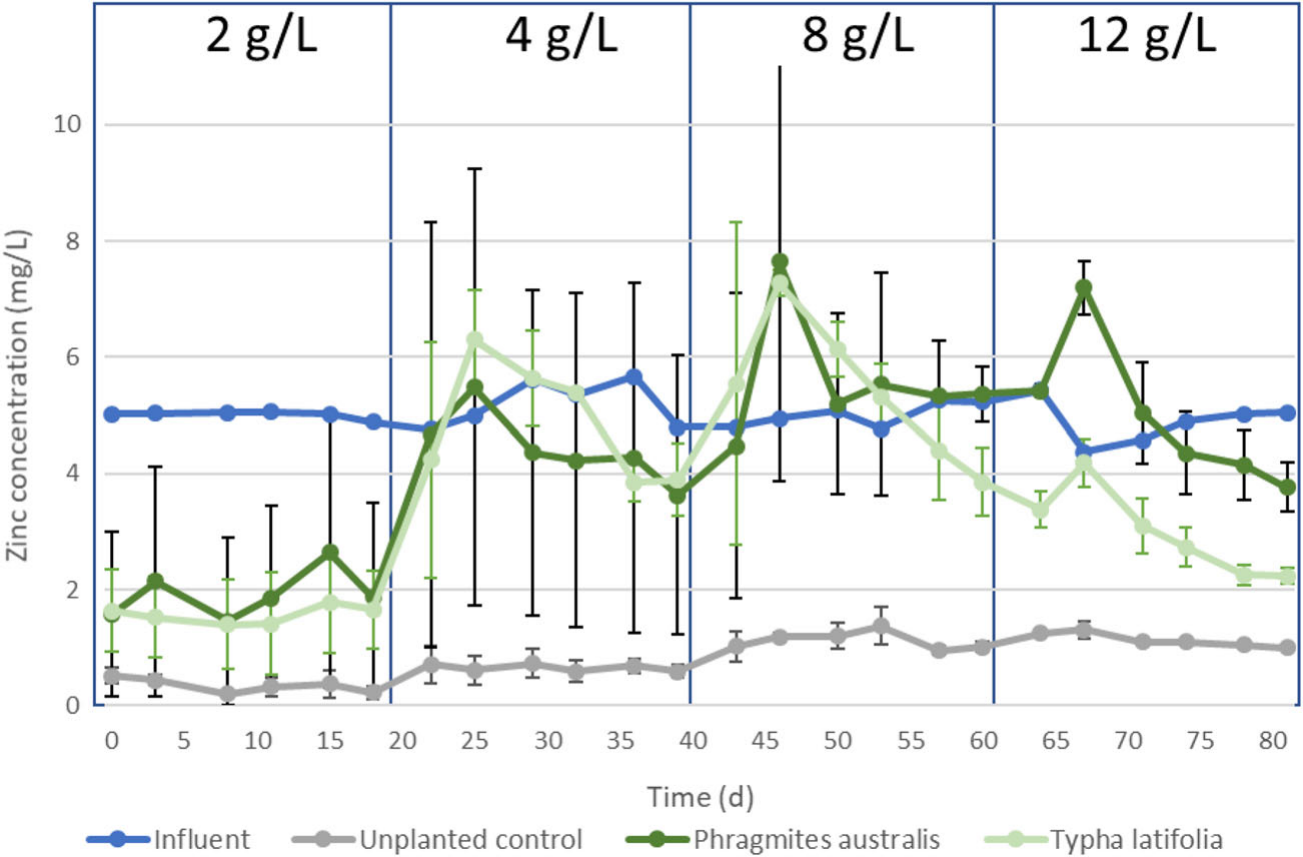
Influent (blue line) and effluent zinc concentrations over time with increasing NaCl concentrations in *P. australis* planted CWs (dark green line), *Typha latifolia* planted CWs (light green line) and unplanted CWs (grey line)

The removal of zinc is substantially impacted by an increase in the NaCl concentration in the *T. latifolia* and *P. australis* planted CWs (Fig. 4). In the unplanted CWs, the effluent concentrations of zinc are not affected by increasing NaCl concentrations, and approximately 80% of the zinc is removed (Fig. 4).

At a NaCl concentration of 2 g/L, the *P. australis* and *T. latifolia* planted CWs have similar effluent zinc concentrations that are higher than those of the unplanted CW (Fig. 4), with substantial deviations in the removal efficiencies of the experimental duplicates of mainly the *P. australis* planted CWs. Evapotranspiration resulted in a higher concentration of zinc in the effluent of the planted CWs, but the evapotranspiration rate cannot fully explain the difference in zinc removal between the planted and unplanted CWs. In addition, the deviation in effluent zinc concentration of the *P. australis* duplicates are not the result of differences in evapotranspiration, since the deviation of the effluent EC in the planted CWs is smaller than the deviation in effluent zinc concentrations (Figs. 1 and 4). The differences in removal efficiency of the planted CWs could be the result of difference in root system development. The removal of zinc in CWs is the result of precipitation in the rhizosphere and on the root surface of wetland plants (Otte et al. 1995) and uptake by the plants (Sheoran and Sheoran 2006). In addition, zinc adsorbs to the CW substrate as result of cation exchange or chemisorption and to clay and organic matter by electrostatic interaction (Sheoran and Sheoran 2006). The lower removal in the planted CWs is probably governed by a rhizospheric decrease of the pH and higher redox conditions by oxygen influx through plants in the planted CWs. The differences in development of the rhizosphere might explain the differences between experimental duplicates. The rhizosphere is able to decrease the pH within 1 cm of the root system by the release of acidic root exudates, and this decreased pH causes changes in metal speciation and solubility (Weis and Weis 2004), and competition for adsorption on the CW matrix between the proton and metal cations. Moreover, oxygen influx through the plant root system might mitigate the establishment of sulphate-reducing conditions in planted systems, which prevents zinc-sulphide precipitation and keeps the Zn ions in solution. Wright and Otte (1999) showed that a decreased pH in the rhizospere increased the concentrations of dissolved zinc near the roots of *T. latifolia*, and the transport of this dissolved zinc to the effluent would result in a lower zinc removal efficiency in the planted CWs. This suggests that more detailed probing of pH, redox and dissolved Zn gradients in the root zones of the CW could shed more light on the actual mechanisms controlling zinc adsorption and desorption in CWs, for which the experimental setup of the present study did not allow for.

An increase in the NaCl concentration from 2 to 4 g/L resulted in a sudden increase in the effluent zinc concentration of the *T. latifolia* and *P. australis* planted CWs to a concentration above the influent concentration of approximately 5 mg/L (Fig. 4). After this increase, the effluent concentration stabilized around 4 mg/L, and never reached the effluent concentration at a NaCl concentration of 2 g/L. A similar increase in effluent zinc concentration was observed with an increase of the NaCl concentration from 4 to 8/L. Such increased effluent zinc concentrations were not observed in the unplanted CWs, indicating that plant-mediated processes are responsible for the observed increased zinc concentrations. The release of zinc from the planted CWs after an increase in the salinity could be the result of the complexation of zinc with root exudates that are released to protect the plant against the increased NaCl concentrations and that increase the zinc mobility (Lutts and Lefèvre 2015). The low increase in the effluent zinc concentration of the *T. latifolia* planted CWs with an increase in NaCl concentration from 8 to 12 g/L, where the *T. latifolia* died (“The influence of salinity on plant growth” section), provides additional proof that the release of Zn at salinity increases at lower salinities is a result of an active plant response mechanisms to increased salinities.

### Reuse of petroleum-industry wastewater in hot arid climates

It can be concluded from the present study that treatment of synthetic mildly saline PI-WW with a NaCl concentration up to 12 g/L in CWs in simulated hot arid climatic conditions results in the removal of the conditioning chemicals benzotriazole and aromatic hydrocarbon benzoic acid from the synthetic PI-WW. However, the present study also shows that increasing salt concentrations of the CW-influent negatively affect the biodegradation of benzotriazole and benzoic acid. Increasing salt concentrations also affect the removal of zinc from the synthetic PI-WW. Zinc is steadily removed from the synthetic saline PI-WW as long as the salinity of the influent is stable, but fluctuating salt concentrations result in spikes of the effluent zinc concentration as result of active plant defence mechanisms. Moreover, evaporation and evapotranspiration in the hot and arid conditions applied in the present study result in an increased EC of the CW treated water.

The increased EC of the CW effluent limits its further reuse options. Al-Ghouti et al. (2019) identified various reuse options for PI-WW: livestock watering, habitat and wildlife watering, fire control, dust control, reuse in the oilfield, cooling water during power generation and irrigation in agriculture. A distinction is made between irrigation of food crops and irrigation of non-food crops that can be used for instance as bio-based fuels or construction material. For non-food crops, the legislative water quality requirements are generally lower (Al-Ghouti et al. 2019). Plant growth can be substantially hindered by too high salt concentrations of the treated PI-WW (Pica et al. 2017). The use of treated saline PI-WW can not only negatively affect the growth of plants directly but can also result in an increased soil salinity and soil sodicity (Echchelh et al. 2018). These processes can irreversibly change the soil structure and significantly lower the soil fertility (Echchelh et al. 2018). Therefore, various countries have guidelines in place with threshold values for the EC, sodium adsorption ratio (SAR) and heavy metal concentrations to which irrigation water has to comply to prevent damage to soil and crops (do Monte 2007; Jeong et al. 2016).

An increased salinity as result of CW treatment does not only limit the applicability of PI-WW for irrigation in many countries, but also its use in industrial processes, such as its use as cooling tower make-up water (Wagner et al. 2018). To make the treated PI-WW suitable for either irrigation or as make-up water, CW treatment can be followed by desalination technologies to obtain appropriate salinities for reuse. Desalination of PI-WW has been studied extensively (Al-Ghouti et al. 2019; Fakhru’l-Razi et al. 2009; Shaffer et al. 2013), and various technologies are capable to lower the salinity of PI-WW to a level suitable for use as irrigation water or in cooling towers. For instance, Mallants et al. (2017) showed that reverse osmosis of PI-WW results in a water source that meets the EC requirements for use as irrigation water. CWs can be applied as a pre-treatment before membrane-based desalination of PI-WW to remove fractions that shorten the lifespan of desalination membranes, such as suspended solids and conditioning chemicals (Wagner et al. 2020c). In conclusion, *P. australis* planted CWs are a suitable option for the treatment of water with a salinity below 12 g/L, and the CW effluent can be reused directly or further desalinated to increase its reuse options. CW treatment and water reuse would be beneficial not only in water-scarce areas but also in areas with stringent PI-WW discharge regulations. Such CWs will not only protect the environment but can also boost the local biodiversity. Practical implementation of CWs for the treatment of high-volume industrial wastewater, such as PI-WW, is described by Stefanakis (2019).

## Electronic supplementary material

ESM 1 (DOCX 1673 kb)
