# Supplementary material for: Effects of salinity on the treatment of synthetic petroleum-industry wastewater in pilot vertical flow constructed wetlands under simulated hot arid climatic conditions
Source: Environ Sci Pollut Res Int. 2020 Sep 1;28(2):2172–81. doi: 10.1007/s11356-020-10584-8 (PMC7785543; doi:10.1007/s11356-020-10584-8)
Supplement: Supplementary file 1 — (DOCX 1673 kb) [file 11356_2020_10584_MOESM1_ESM.docx]

**Supplementary info to**

**Effects of salinity on the treatment of synthetic petroleum-industry wastewater in pilot vertical flow constructed wetlands under simulated hot arid climatic conditions**

*Thomas V. Wagner^ab^, Fatma Al-Manji^a^, Jie Xue^a^, Koen Wetser^a^, Vinnie de Wilde^a^, John R. Parsons^b^, Huub Rijnaarts^a^, Alette Langenhoff^a^*

^a^ Department of Environmental Technology, Wageningen University, P.O. Box 17, 6700 EV Wageningen, The Netherlands

^b^ Institute for Biodiversity and Ecosystem Dynamics (IBED), University of Amsterdam, P.O. Box 94248, 1092 GE Amsterdam, the Netherlands

**Content:**

**Figure S1** – Development of plants under increasing salinities.

**Figure S2** – pH of influent and effluent over time with increasing NaCl concentrations

**Figure S1.**


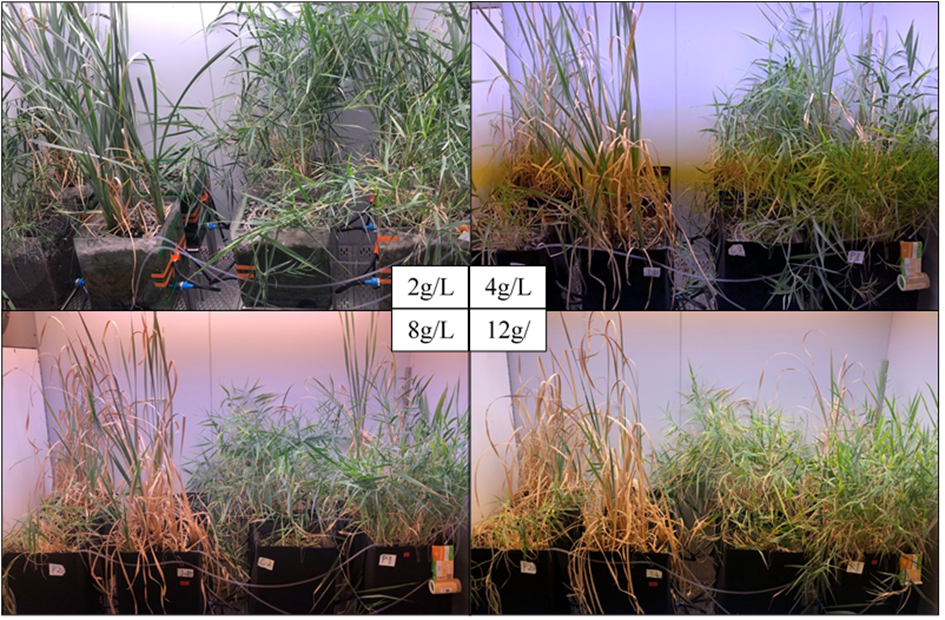


Figure S1. The development of Typha latifolia (left) and Phragmites australis (right) with different concentrations of NaCl.

**Figure S2.**


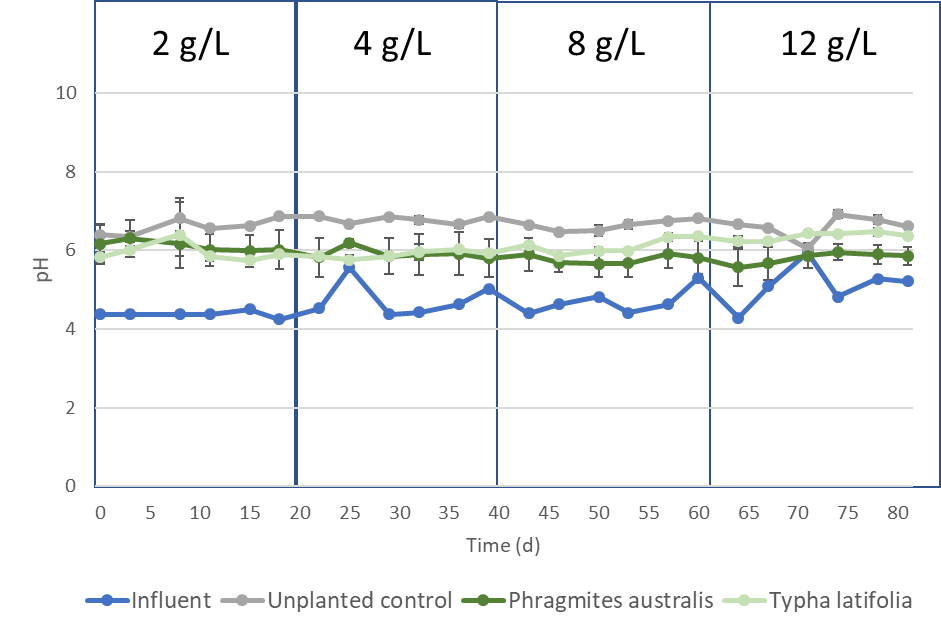


Figure S2. pH of influent and effluent over time with increasing NaCl concentrations
